# Instagram shared tasks in reducing speech anxiety among international students learning Turkish

**DOI:** 10.3389/fpsyg.2026.1775400

**Published:** 2026-03-17

**Authors:** Ali Uzun

**Affiliations:** Turkish Language Education, Kahramanmaraş Sütçü Imam University, Kahramanmaraş, Türkiye

**Keywords:** Instagram, mixed research method, social media, speech anxiety, Turkish

## Abstract

**Purpose:**

The aim of this study is to examine the effect of sharing Turkish speaking tasks on Instagram on the Turkish speech anxiety of international students learning Turkish at the B2 level, using a quasi-experimental intervention design, and to elaborate on the students' perceptions and experiences of the process through qualitative data.

**Methodology:**

This research used an explanatory sequential design within a mixed-methods approach. The study involved 39 international students learning Turkish at the B2 level in Turkey. Out-of-class speaking (interview) tasks and their sharing on the institutional Instagram account lasted for 6 weeks with 17 students in the experimental group. Quantitative data were collected using pre- and post-tests of the Anxiety Scale for Speech Anxiety. Qualitative data were obtained through semi-structured interviews to explore in depth the experimental group students' perceptions, experiences, and justifications regarding the activity and implementation process, and to develop explanatory themes related to the background of the quantitative findings. Quantitative data were analyzed using mixed ANOVA, and qualitative data were analyzed using thematic analysis. Quantitative and qualitative data were then integrated and interpreted.

**Results:**

The quantitative findings of the study show that EG, who participated in Instagram-based conversation tasks, had a statistically significant advantage over CG, who performed conversation activities based on the current curriculum, in both speech anxiety and the development of speech skills. Furthermore, consistent with the task-based foreign language learning principle, which is the theoretical basis of the study, it was found that students' Instagram posts were made in the context of assigned tasks and that this had a positive effect on their speech anxiety. The research offers insights into rapidly growing social media trends regarding the creative use of technological innovations for language learning.

## 1 Introduction

Social media tools, which are now an integral part of mobile technologies, have become inseparable from daily life and are widely used by new generations, especially university students. Education, particularly with the integration of social media and web-based platforms, is undergoing significant changes across academic disciplines, making their use increasingly inevitable and important for almost everyone (Bui et al., 2023; Chau and Bui, 2023). Social media tools such as Instagram, X, and YouTube are actively used in the process of learning a foreign language, one of these disciplines. Foreign language learners also experience anxiety about speech the target language, often defined as fear or anxiety about communicating due to the stress of speaking the new language, usually in a classroom setting or in front of peers (Shadiev et al., 2024).

Instagram, widely used for education and in various ways, is a preferred social media platform because of its accessibility, user-friendly interface, and diverse content. With over 46 million users in Turkey, Instagram is the second most preferred social media platform (Taştekin, 2023). As students are constantly exposed to social media platforms like Instagram in foreign language learning environments, it is natural for them to use these tools to go beyond the limitations of traditional teaching and address various needs (Barrot, 2020). Instagram use also has psychological effects, particularly on young people. (Shahouzahi Moghaddam 2025) states that Instagram can be a valuable educational tool when used appropriately, that its use extends beyond simple communication, and that many users view Instagram as a way to reduce daily stress. In this context, it is evident that task-based conversation applications and Instagram can be effective tools for reducing students' anxiety about speaking in Teaching Turkish as a foreign language (TFL), serve as platforms for production and benefit in teaching environments rather than being consumption-oriented, and make significant contributions to the literature, particularly regarding TFL.

The limited number of studies on the use of social media in foreign language teaching is notable (Krutka and Carpenter, 2016; Taskiran et al., 2016; Vivakaran and Neelamalar, 2018; Maulina et al., 2021; Devana and Afifah, 2021). (Lee 2023) conducted a study highlighting the potential of Instagram and TikTok for language learning. (Argin and Çetinkaya 2019) found that learners who used social media to learn a foreign language achieved outcomes such as active participation, lesson support, and learning through videos. Social media applications such as Instagram and Twitter allow learners to actively use the language they are learning in their daily lives (Yilmaz, 2025). Some studies have suggested that Instagram's interactive image-sharing features can be used in foreign language learning (Al-Ali, 2014; Purnama, 2017), and that social media tools are effective in developing skills and components such as communicative competence and cultural awareness in foreign language learners (Vásquez, 2014; Reinhardt, 2019). Most studies focus on the use of social media in general in language teaching or foreign language teaching. Only a limited number of studies (e.g., Al-Ali, 2014; Aloraini, 2018; Erarslan, 2019; Gonulal, 2019; Rizal and Farikhah, 2021; Kaviani, 2022) are directly related to the use of Instagram, mainly for EFL, in foreign language teaching. Although there are many studies on the intensive use of social media applications in language teaching, the lack of experimental research on Instagram in teaching a foreign language other than EFL is notable.

In this context, examining the use of Instagram in TFL teaching through extracurricular activities and its effect on students' anxiety about speaking Turkish addresses an important gap from both theoretical and experimental perspectives. Furthermore, the increase in the number of international students in Turkey from 48,183 to 337,073 over the past decade, along with the country's high ranking in attracting international students in Europe (YÖK, 2025), increases the need for innovative and scalable teaching designs in TFL.

This study focuses on a multi-stage social media interaction cycle that integrates Instagram into the speaking process of students in TFL. The research examines the effect of Instagram-shared speaking tasks on the speech anxiety of international students learning Turkish at the B2 level using a quasi-experimental intervention design. It also presents students' perceptions and experiences regarding the use of Instagram in TFL through qualitative data.

In this regard, the study (i) contributes contextually by providing experimental evidence on the development of Instagram-supported speaking skills within the TFL context, (ii) contributes methodologically by testing the cycle of social media-supported multi-stage speaking activities using a mixed-method approach, and (iii) addresses speech anxiety within the framework of Task-Based Language Learning (TBLL) theory, generating theoretical insights regarding the relationship between Instagram-supported Turkish speaking activity sharing and the dimensions of autonomy and competence. Within this framework, the study sought answers to the following research questions:

1. How do Instagram shared conversation tasks affect TFL students' anxiety about speaking Turkish?
2. What are TFL students' experiences with the contributions, limitations, and extracurricular use of activities shared on Instagram?

### 1.1 Background and theoretical framework

#### 1.1.1 Anxiety about speaking a foreign language

Foreign language anxiety (FLA) generally arises from the complex interaction of factors such as fear of negative evaluation, communication anxiety, and self-consciousness (MacIntyre and Gardner, 1994). FLA typically appears during spoken communication, resembling feelings like stage fright or public speaking anxiety, and hinders active participation in language communication (Carlisle et al., 2024). Learners of a new language should not only study the target language theoretically but also have the confidence to use it in real-life situations where it is spoken. Developing speaking skills in foreign language teaching is a highly challenging and complex process (Sönmez and Güneş Özden, 2024). Stressed foreign language learners describe speaking in the target language as the most intimidating skill (Yalçin and Inceçay, 2014). According to (Gass et al. 2020), foreign language anxiety is one of the most common emotions experienced by these learners. Although this anxiety is initially a major obstacle, educators and practitioners can minimize it, helping students overcome a significant barrier. It is possible to reduce the anxiety of individuals who experience foreign language speaking anxiety by engaging certain psychological mechanisms. One such mechanism, peer evaluation and frequent error correction feedback, helps individuals improve their speaking skills and reduce their anxiety about speaking a foreign language, regardless of their initial anxiety level (Geçkin, 2020). Furthermore, interventions aimed at coping with speaking anxiety require the creation of environments that expose students to speaking opportunities and provide a sense of support for practice (MacIntyre and Gregersen, 2012). It has been observed that students experiencing speech anxiety show a gradual decrease in their anxiety levels, especially when exposed to performance situations (Finn et al., 2009). In addition, removing the threat of evaluation in feedback given to students' foreign language speaking activities has a positive effect on reducing speaking anxiety. Providing supportive and inclusive classroom environments where foreign language learners can express themselves comfortably without fear of judgment, along with positive reinforcement and encouragement, can help alleviate anxiety (Carlisle et al., 2024). Creating practice environments where students can comfortably express themselves in speaking—the skill with the highest anxiety—is an important step in overcoming speaking anxiety. Communication with native speakers of the target language especially increases students' speech anxiety in foreign language learning. As students experience less anxiety, they move closer to success; as they achieve success, their anxiety decreases. (Yalçin and Inceçay 2014) found that the feeling of success significantly affects anxiety, and students become more relaxed as their success rate in completing tasks increases. Students should participate willingly and actively in extracurricular activities. For this reason, using Instagram TFL, one of the mobile-supported language learning tools that attracts the interest of young foreign language learners and is frequently used by them, as a tool to reduce speech anxiety and develop speaking skills among learners was considered important and tested. In addition, TFL students are expected to increase both their academic and Turkish speaking self-efficacy by completing the assigned tasks. Self-efficacy is defined by (Bandura 1993) as students' assessments of their ability to complete a specific task and achieve academic goals. Furthermore, (Wang et al. 2021) stated that students with high self-efficacy have low anxiety, high academic achievement, and greater enjoyment of learning. Instagram serves as an entry point and preparatory environment for socialization, especially for those who lack confidence and experience communication anxiety when learning a foreign language. When someone learning a foreign language follows or participates in Instagram-supported speaking activities, they may feel psychologically relieved. Therefore, Instagram, as a mobile-supported language learning tool, can be considered an important support and preparatory environment for those who experience foreign language speech anxiety. This is because Instagram introduces foreign language learners to real-life language usage contexts while also encouraging them to adapt to global language norms (Jerome and Sathya, 2024).

#### 1.1.2 Mobile-assisted language learning and Instagram

With technological advancements, computer-based learning tools have become increasingly accessible and are now available as portable devices that anyone can easily use. Mobile-Assisted Language Learning (MALL) refers to learning with the help of handheld technologies such as cell phones, PDAs, iPods, iPads, and similar devices (Lim and Felix, 2021; Kurt Dizbay, 2023; Ali, 2014; Davie and Hilber, 2015). In addition to MALL-focused tools, some social media platforms (Facebook, X, TikTok, Instagram) have recently been incorporated into MALL (Gonulal, 2019). Facebook (Alm, 2015; Kabilan et al., 2010; Mitchell, 2012) and X (Lomicka and Lord, 2012; Solmaz, 2017; Taskiran et al., 2018) have been shown to be important tools for facilitating interaction among learners in language or foreign language learning. The integration of MALL technology into language education has prompted researchers to examine the potential effects of MALL tools on the language development of academic language learners (Tavassoli and Beyranvand, 2023). In this context, studies have explored the effective use of social media tools in language teaching within the scope of MALL. Instagram, as one of these social media tools, is an effective platform for task-related activities, foreign language teaching, and student interactions (Mansor and Rahim, 2017). However, despite Instagram being a popular social networking platform with significant MALL potential, the relatively small number of studies focusing on Instagram makes this research important for its contribution to the literature.

#### 1.1.3 Task-based language learning theory

TBLL uses tasks as a central component in the language classroom because they provide better contexts for activating student acquisition processes and promoting second language learning (Dorathy and Mahalakshmi, 2016). TBLL, also known as Task-Based Instruction, has been a prominent and extensively researched area in language pedagogy and second language acquisition since the 1980s. To address the research questions, this study adopts the concept of task-based language learning to determine the opportunities Instagram provides and how foreign language learners use them. The TBLL approach was first developed in 1982 in the Bangalore research report by Prahbu in South India (Göçer and Karadag, 2020). TBLL approach differs from traditional teaching methods and extends the communicative approach (Ellis, 2003). This approach, which uses interactive and student-centered teaching methods, helps students learn a foreign language by completing tasks both inside and outside the classroom and can also familiarize them with real-life communication (Göçer and Karadag, 2020). Similarly, (Richards and Rodgers 2001) state that task-based activities, such as assigning roles and role-playing in the classroom, can improve students' speaking skills by engaging them in communication and interaction. Furthermore, a study by (Mansor and Rahim 2017) revealed that Instagram is an effective social media tool for student communication, particularly in task-related activities.

In foreign language teaching, adopting new approaches tailored to the characteristics of Generation Z is crucial. This generation's digital literacy and affinity for technology require a reassessment of educational methods (Gözüküçük, 2024). The current educational environment emphasizes the use of digital technology and social media tools in teaching and learning (Rautela et al., 2024). Therefore, it is necessary to adapt to the diverse needs of students (Klimova, 2014; Fu et al., 2025), ensure their collaborative participation both inside and outside the learning environment (Hao, 2016), and motivate them to engage in their studies (Fathi and Rahimi, 2022). Today, the integration of mobile technology into language education has become a daily phenomenon (Zaki and Yunus, 2015). As today's learning environments are equipped with technology and digital tools, and foreign language learners use these as teaching materials, social media platforms such as Instagram, which offer interaction and appeal, can serve as important educational resources. Foreign language learners can also reduce their anxiety about speaking by becoming accustomed to real-life communication through out-of-class assignment applications. This study will provide educators considering the use of Turkish speaking tasks shared on Instagram in their teaching processes with a roadmap based on participant feedback, aimed at creating environments where students can acquire everyday language used in real life, which is more natural than classroom language.

## 2 Research methodology

### 2.1 Research design

This study uses an explanatory sequential design within a mixed-methods approach, involving data collection and analysis to examine the effect of one variable (posting speaking tasks on Instagram) on another variable (foreign students' anxiety about speaking Turkish). In an explanatory sequential design, quantitative data are collected and analyzed in the first stage, followed by a qualitative stage designed to explain and deepen the quantitative findings (Creswell and Plano Clark, 2018; Ivankova et al., 2006).

**Figure 1:**
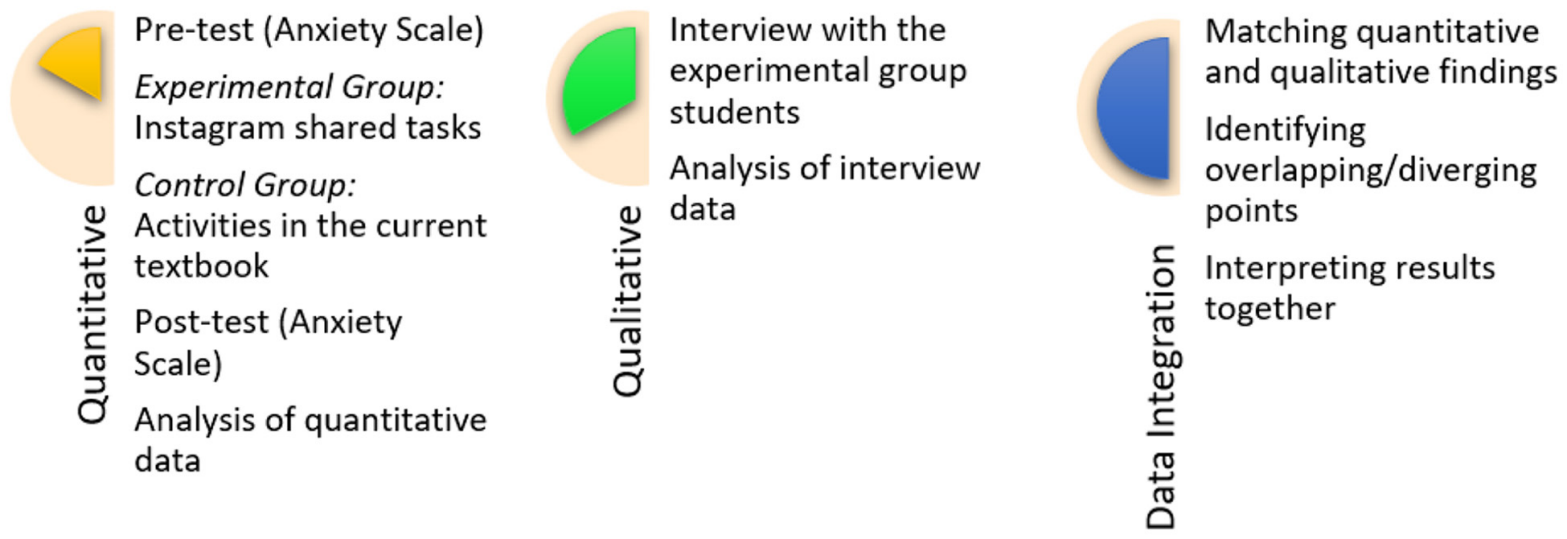
Steps of the mixed quantitative–qualitative method used in the study.

The quantitative component of the study used a pretest–posttest control group quasi-experimental design to examine the effect of Instagram shared conversation tasks on Turkish conversation anxiety. The aim was to statistically test the experimental effect by comparing pre- and post-intervention scores. In the qualitative component, semi-structured interviews were conducted only with students in the experimental group (EG) to examine their perceptions, experiences, and justifications related to the implementation process in depth. Explanatory themes related to the background of the quantitative findings were developed (Draucker et al., 2020). Quantitative and qualitative data were integrated at the levels of design, method, and interpretation, as specified by the explanatory sequential mixed-methods design. Comprehensive meta-inferences regarding the effectiveness of Instagram shared conversation activities were developed, particularly through the use of “connecting” and “merging” integration strategies (Fetters et al., 2013). This approach, which involves collecting and analyzing quantitative and qualitative data together, is grounded in the robust methodological framework and principle of integrating multiple data sources proposed by (Phakiti 2014) in second language research. The quantitative–qualitative mixed methods steps followed in the study are summarized in Figure 1.

### 2.2 Participants

The study was conducted in 2025 with 39 international students learning Turkish at the B2 (Intermediate) level at a state university in Turkey. Participants were international students from two classes at the university where the research was conducted, selected through convenience sampling. This method is commonly used in classroom-based second or foreign language research, allowing the researcher to form a sample based on criteria such as geographical proximity, accessibility, and voluntariness (Dörnyei, 2007; Mackey and Gass, 2022). One class was designated as the EG and the other as the CG. Student names were kept confidential during data analysis and presentation of findings; instead, participants were coded as S1, S2, S3, and so on. The EG included 13 males and 4 females, while the CG included 10 males and 12 females, all aged 17–22. Participants were from various countries, such as Libya, Burkina Faso, Afghanistan, Pakistan, Mali, Senegal, Yemen, Zimbabwe, Ethiopia, Benin, Tanzania, Guinea, Congo, Gabon, Somalia, Equatorial Guinea, Niger, Ivory Coast, Indonesia, and Mauritania, representing a multilingual and multicultural learner profile. Their languages included Arabic, French, English, Spanish, Persian, Somali, Wolof, Songhay, Amharic, Yoruba, and Swahili. Most students had less than 6 months of Turkish learning experience, with only a few having 6 months or more, indicating that the sample consisted of B2-level international students enrolled in an intensive, short-term teaching program.

### 2.3 Data collection tools

In this study, data were collected in two consecutive stages as required by the explanatory sequential mixed design. First, quantitative data were collected using a single instrument administered to both groups before and after the experimental intervention. The quantitative data collection tool was the “Anxiety Scale for Speech Anxiety,” developed and applied by Özdemir (2013) and re-tested for validity and reliability by (Zhu 2023). The Cronbach's alpha reliability coefficient for this scale was calculated as 0.80 by Özdemir (2013) and 0.89 by (Zhu 2023). The optimal Cronbach's alpha range accepted in the literature is 0.7–0.9 (Büyüköztürk, 2007; Sencan, 2005). (Hair et al. 2019) state that “The CR coefficient is a coefficient that takes values between 0 and 1, and values greater than 0.7 are expected.” In this study, the pre-test reliability coefficient for the scale was calculated as 0.75, while the post-test reliability coefficient was 0.84. Based on these findings, it is understood that the scale used in the study maintained its consistency over time and that the measurements were reliable.

Secondly, qualitative data were collected using a “Semi-Structured Student Opinion Form” prepared by the researcher with seven items and submitted to field experts (one professor and two foreign language instructors experienced in TFL) for their feedback. The items in the draft form were organized around themes such as the possible effects of Instagram-shared activities on Turkish speech anxiety and Turkish speaking skills, the limitations encountered, and trends in out-of-class usage. These themes were identified based on findings from recent studies on the use of social media in foreign language teaching (Argin and Çetinkaya, 2019; Lee, 2023).

To ensure the content validity of the interview questions, the draft was submitted for expert review. Experts were asked to evaluate the questions for scope appropriateness, clarity and comprehensibility, alignment with target concepts, and non-directiveness. Based on their recommendations, overlapping items were combined, the wording was simplified, and a final 5-item form was created to capture the experiences of students participating in Instagram shared conversation tasks.

During data collection, interviews were conducted using a pre-prepared semi-structured interview guide (Appendix 2). All participants were asked the same basic questions in the same order, and only neutral probing questions were used to elicit detailed responses. This standard practice ensured that the interviews progressed appropriately and consistently examined the intended question structures, thereby strengthening the credibility of the qualitative findings. Furthermore, the planned execution of the interview process contributed to the internal validity of the research and its replicability in different contexts (Arslan, 2022; McCracken, 1988; Sekaran, 2002).

### 2.4 Application process

The B2 level in TFL instruction lasts for 6 weeks. Therefore, the application process was set for 6 weeks. In the first week of the application, the researcher (instructor) provided information about the “Anxiety Scale for Speech Anxiety” to both the experimental and control group students, and this scale was administered as a pre-test during two speaking lessons (80 min). In addition, the experimental group was informed about TBLL during this week. This ensured that a standard was established between the groups in accordance with the tasks in the application guidelines. In the final week, the pre-test administered to both groups was repeated as a post-test during the speaking lesson (80 min) by the researcher.

In both the experimental and control groups, the speaking course—one of the four core language skills (reading comprehension, writing, speaking, listening)—was taught by the same instructor for 2 h per week (80 min) as part of the TFL curriculum. In the experimental group, speaking courses were supplemented with speaking activities conducted by students with native Turkish speakers. Additionally, students' extracurricular speaking activities were posted on Instagram, and each group's posts were evaluated. These evaluations generally consisted of peer and teacher feedback on the speaking (interview) activities of group members. Furthermore, the researcher guided the application through the social media group during extracurricular time, without any space or time constraints. In the control group, the speaking class was conducted weekly as traditional in-class speaking activities, following the existing curriculum and covering the same speaking topics and duration as in the experimental group.

During the implementation process, it was decided to prepare an activity implementation guideline by first reviewing the literature on foreign language anxiety and the use of social media and Instagram in language teaching. This guideline was prepared separately for each week and served as a guide for students and practitioners. The activity implementation guidelines were developed by the researcher to include speaking topics covered in the curriculum and textbook within the scope of TFL learners' speaking skill objectives. The guidelines include instructions such as the topic of the week's activity, examples of expressions for starting and ending conversations with Turkish peers, conversation duration, interview questions, suggestions for the location of the activity, and video submission (see Appendix 1). EG teams were then formed. It is important to use strategies that help teams organize their group work effectively, improve their planning processes (Yilmaz et al., 2020), and increase students' participation in learning activities and collaborative processes (Noguera et al., 2018). Students conducted interviews with their peers at the university where the study was conducted or with native Turkish speakers in that city on predetermined topics. Participants randomly selected the people they interviewed but obtained their consent beforehand by informing them that these conversations would be published on Instagram. As some individuals did not agree to this, the interviews were not conducted with them; they were only conducted with Turkish individuals who gave permission for their conversations to be published on Instagram. The interview process was recorded on video by one of the group members. As stated in the “Implementation Guidelines,” care was taken to ensure that the video recordings were at least 2–3 min long. The weekly lesson schedule mentioned in the activity guidelines is as follows:

During the first week of the study, quantitative scales were administered to both groups. In the same week, heterogeneous project groups were formed with the EG students, and each group was assigned a number. The oral communication and speaking topics included in the B2 level curriculum were determined. TFL B2 lessons began simultaneously in the experimental and control groups.

In the second week of the study, EG students began working in their project groups, dividing tasks according to the activity implementation guidelines. They performed the first activity, “Conversation About Vacation,” with native Turkish speakers outside of class. The video-recorded interviews were published on the university's TÖMER Instagram account by the relevant administrator after being reviewed by the researcher.

EG students, who also used a collaborative learning approach, held meetings and discussions about project topics in line with the weekly guidelines. In keeping with the principle of learner autonomy, students planned the activities themselves. During this process, the researcher acted as a guide and provided support by communicating with students via social media applications.

**Figure 2:**
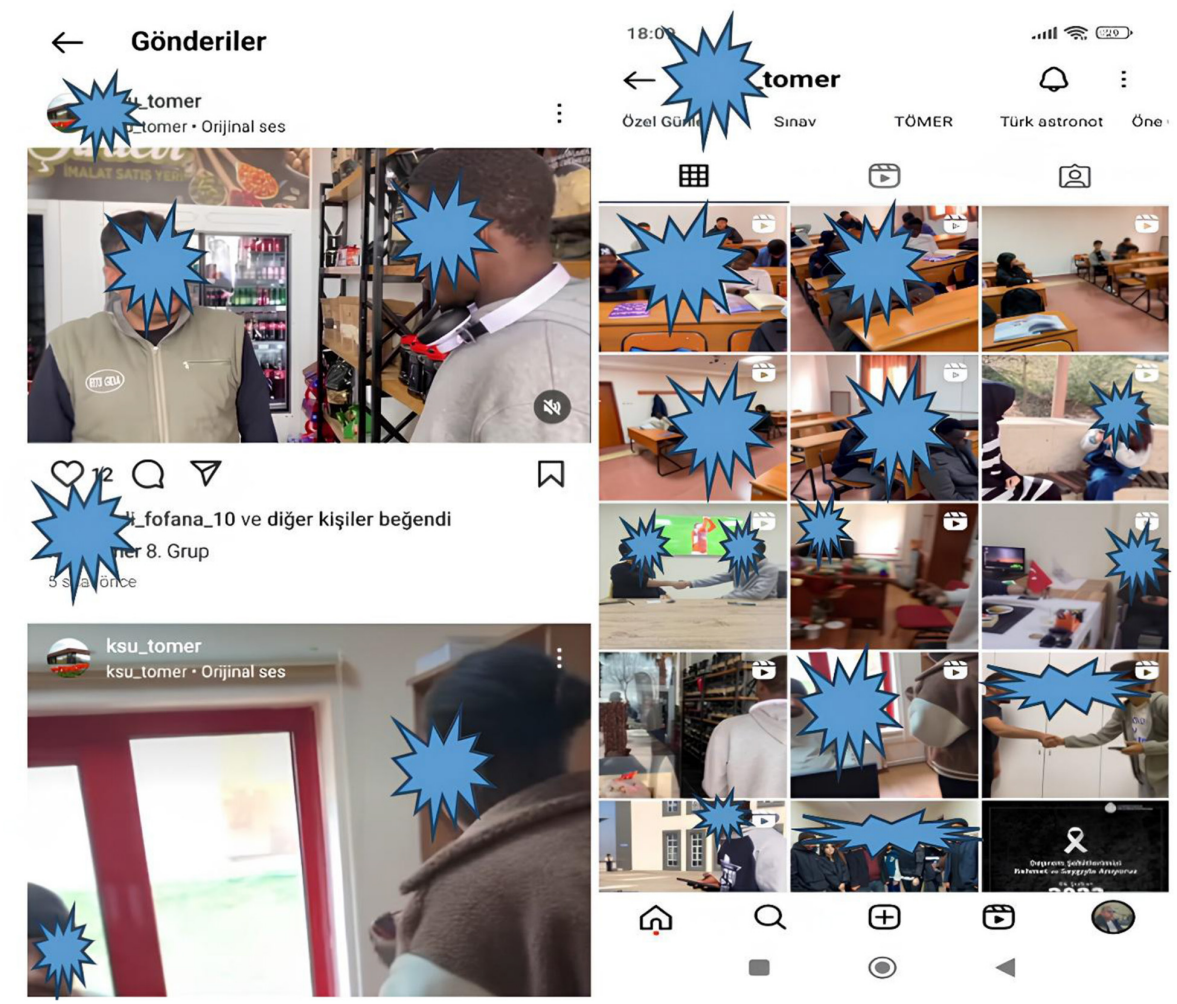
A section of the event application and Instagram post.

Additionally, EG students conducted the “Discussion on Social Media Use” activity with individuals whose native language is Turkish. These events were also broadcast on Instagram. In the study, the third event, “Discussion on Shopping Preferences,” took place in the fourth week, and the fourth event, “Discussion on Technology,” occurred in the fifth week. These events were also broadcast on the TÖMER Instagram account within the university (Figure 2).

During this process, the consistency of the research and the quality of the intervention were maintained through a structured monitoring and evaluation system. An education lecturer, other than the researcher, monitored the weekly activities. The activities were then reviewed with the practitioner and the external observer via the Instagram account, and weekly observation notes were recorded. For example, in the second week, the researcher observed the obstacles and difficulties students encountered during their first interview activities; however, due to learner autonomy, no direct intervention occurred. Later, all groups shared similar incidents they had experienced during a class meeting, and the researcher proposed solutions to these situations. This provided students with learning environments where they could use their decision-making and problem-solving skills. Below is a sample observation note from the second week's application:

2. Weekly Observation Note (Researcher):

*Students planned how to conduct their interviews according to the application guidelines by collaborating in self-formed groups. They appeared somewhat nervous during their first interviews. Some Turks did not want to be interviewed because the students were foreigners, which made the students somewhat anxious. All students actively participated in the activities. Later, they sent the recorded interview videos to the researcher. In subsequent lessons, students provided criticism, congratulations, and comments on the videos posted on Instagram, thus conducting an application critique. Overall, the students' attitudes were observed to be positive*.

In the sixth and final week of the study, the final test was administered to the experimental and control group students during two speaking class hours. Qualitative data were collected using a “Semi-Structured Interview Form” to evaluate the experiences of the experimental group students regarding the process of sharing speaking tasks on Instagram.

**Figure 3:**
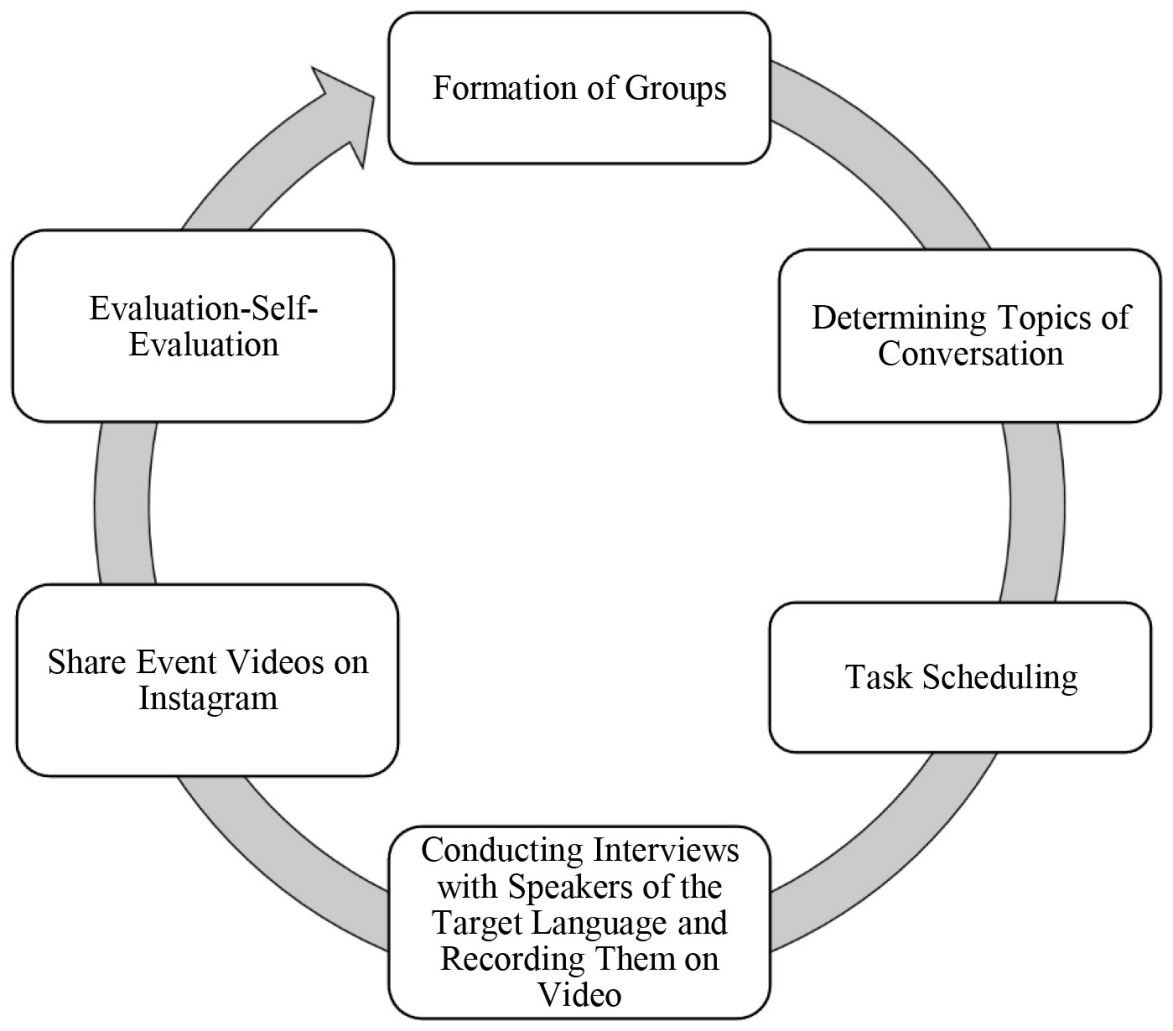
Weeks 1–6: Instagram shared conversation task cycle (EG).

In the control group, the same learning outcomes and speaking topics were covered in the same amount of time, using only the speaking activities in the B2-level textbook and teacher-student interaction in the classroom. The goal for both the experimental group and the control group was for students to acquire B2-level Turkish speaking skills by the end of this period. Thus, the fundamental difference between the two groups was the publication of task-based speaking video recordings on Instagram, which was implemented only in the experimental group. This activity cycle in the EG is shown in Figure 3.

This intervention study, based on conversation tasks shared on Instagram for foreign language learners, is theoretically supported (Mahapatra, 2024; Polakova and Ivenz, 2024).

### 2.5 Data analysis

The quantitative and qualitative data collected in the study were categorized and analyzed using appropriate methods in separate stages. The data were analyzed according to the basic stages of data analysis (Phakiti, 2014). In the first stage of the data analysis process, conducted with SPSS, all data from the study group were checked and prepared for analysis. In the second stage, the data obtained from the scales were systematically coded, assigned numerical values, and entered into SPSS. The accuracy of these data was confirmed using a boxplot graph, completing the third stage. In the fourth stage, reliability analyses were performed on the pre-test and post-test data from the scales. In the fifth stage, analyses based on the overall mean of the scale were conducted. In the sixth stage, a normality test was conducted on the quantitative data set to determine the appropriate tests for data analysis. To assess the normality assumption in more detail, the Shapiro–Wilk test was applied to the research variable because the sample sizes were below 50 (Ghasemi and Zahediasl, 2012; Kim, 2013). The Shapiro–Wilk significance values were greater than 0.05 (*p* = 0.793–0.491). This result indicates that the speech anxiety scores did not show a statistically significant deviation from a normal distribution.

After this assumption was met, a separate 2 (Group: experimental, control) × 2 (Time: pretest, posttest) mixed ANOVA was conducted for the dependent variable to examine the change in Turkish speech anxiety pretest–posttest scores between the experimental and control groups. This analysis tested (a) the main effect of time (overall change before and after the intervention), (b) the main effect of group (experimental–control difference), and (c) the Time × Group interaction effect (whether the experimental procedure produced a different change compared to the control group). Mixed ANOVA was chosen because it allows testing both between-group and time-dependent change within the same model and is commonly used in pretest–posttest controlled experimental designs (Field, 2018). The significance level was set at 0.05 for all analyses.

In the analysis of qualitative data, written responses to the interview form were transferred to an electronic format and imported into MAXQDA software. During the coding process, an initial code list based on the research questions was created. New codes derived from the data were added through repeated readings of the text, and similar codes were grouped under higher-level categories and themes. The principles of thematic analysis were followed throughout this process. Particular attention was given to dimensions such as the contributions, limitations, usage preferences, and suggestions perceived by students regarding Instagram-shared conversation activities in relation to their speaking skills and speech anxiety (Braun and Clarke, 2006; Kuckartz and Rädiker, 2019). The resulting themes were then compared with quantitative findings, completing the holistic interpretation process required by the explanatory sequential design. Furthermore, to ensure verifiability in the study, the data was structured directly from participants' statements to minimize researcher bias and ensure objective data analysis (LeCompte and Goetz, 1982).

To enhance the reliability of the qualitative analysis, the coding process was conducted independently by a faculty member (associate professor of educational sciences) other than the researcher. To ensure inter-coder reliability, 25% of the data set selected from the sample was coded separately by an independent subject matter expert. The codes obtained were compared, and the agreement rate was calculated using the (Miles and Huberman 1994) reliability formula [Reliability = Agreement/(Agreement + Disagreement)], yielding an agreement rate above 85%. Any conflicts that arose during the coding process were discussed among the coders and resolved through consensus. For example, one participant's statement, “Doing activities with my friend,” was coded as “collaboration” by one coder and “peer influence” by another. In this case, the reasons given by both coders were discussed; it was decided that the statement emphasized both social and cognitive aspects, and the code was combined as “collaboration.” Such consensus processes ensured that the themes were developed in a comprehensive and consistent manner.

## 3 Results

### 3.1 Findings related to the first research question

Before analysis, the parametric test assumptions for the data were carefully examined. According to the results of the Levene test, the assumption of homogeneity of variances between groups was met for the “Final test” measurement [*F*_(1, 37)_ = 1.99; *p* = 0.166]. Although the Levene test indicated a marginal violation of variance homogeneity in the pre-test measurement [*F*_(1, 37)_ = 5.03; *p* = 0.031], the mixed ANOVA was conducted. This decision was based on: (1) the experimental and control group sizes being equal or nearly equal, (2) the Q-Q plots confirming the normality assumption for the data distribution, and (3) the robust nature of mixed ANOVA against homogeneity deviations in balanced designs, as noted in the literature (Blanca et al., 2017).

Since analysis of variance is a powerful statistical procedure, the homogeneity assumption can be violated for small effects if normality is ensured (Büyüköztürk, 1997). In such cases, if group variances are expected to be unequal, sample sizes should be kept as close as possible (Howell, 2010). ANOVA models are resistant to assumption violations even in small samples; it is stated that if the compared groups are normally distributed and group sizes are equal, violation of homogeneity will not cause serious problems (Wilcox, 2012).

**Figure 4:**
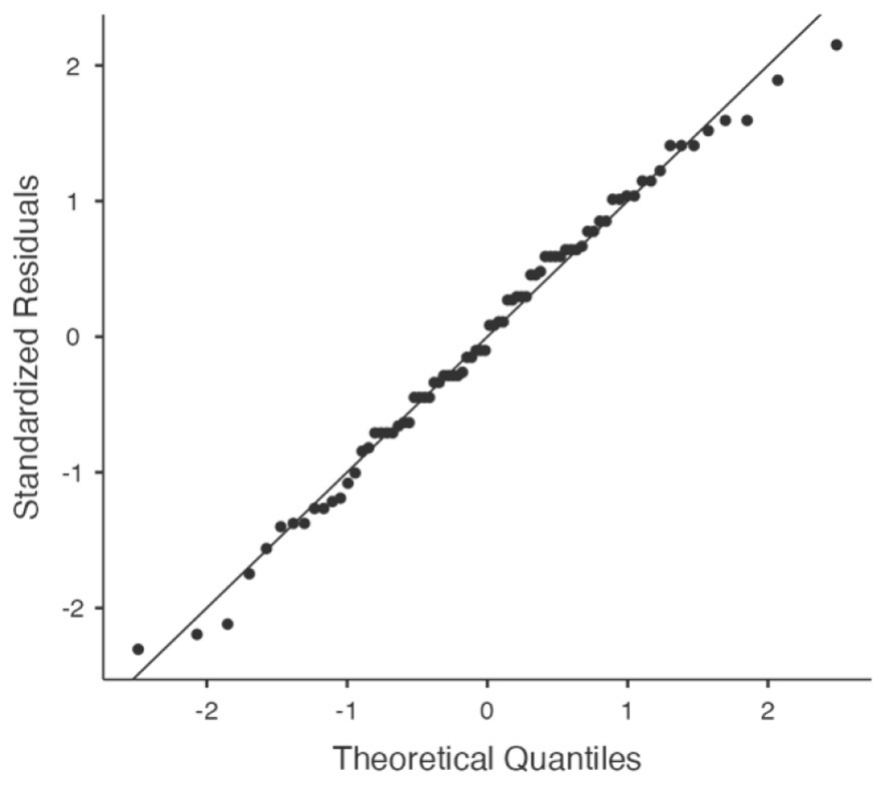
Q-Q Plot analysis visual.

To confirm that the deviation in question does not compromise the validity of the analysis, the Q-Q Plot (Quantile-Quantile Plot) in Figure 4 was examined. The observed values clustered largely along the theoretical normal distribution line, and no significant distortion from outliers was found. This strong visual evidence confirmed that parametric tests can be applied within the assumed confidence intervals despite the marginal deviation in the Levene test.

Descriptive statistics for the pre-test and post-test speech anxiety scores of the experimental and control groups are presented in Table 1.

**Table 1:** Descriptive statistics for speech anxiety.

**Group**	Pre-test		Post-test	
	$\bar{\text{x}}$	**SD**	$\bar{\text{x}}$	**SD**
Experiment	54.41	5.45	41.41	7.272
Control	53.55	3.48	51.82	5.586

**Figure 5:**
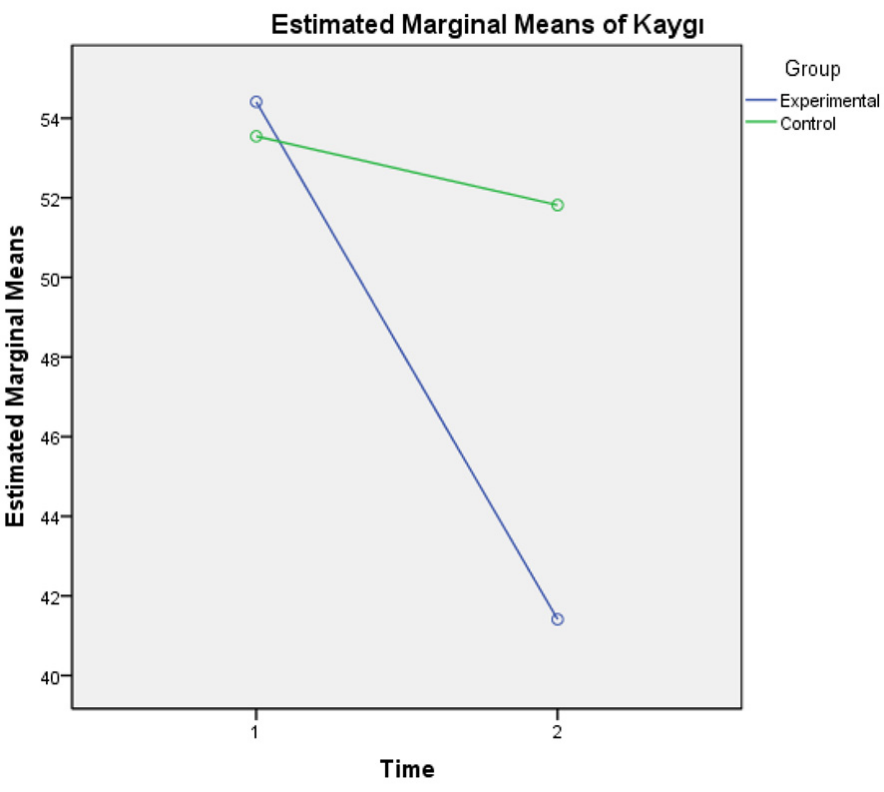
Estimated marginal means for speech anxiety scores.

When examining the pre-test averages, the speech anxiety scores of the EG (x = 54.41; SD = 5.45) and the CG (x = 53.55; SD = 3.48) are quite close, indicating no significant difference between the groups before the intervention. However, the post-test results show that the change between the two groups differed. The EG average score dropped to 41.41, indicating a significant decrease in anxiety levels, while the CG average fell to 51.82, but anxiety did not decrease significantly. The standard deviation values (experimental SD = 7.27; control SD = 5.59) were relatively close at both measurement times, suggesting that the distributions within the groups were similar and that the observed differences were mainly due to changes in the mean level. This is also clearly shown in the graph of the estimated marginal means (Figure 5).

The results of the repeated measures ANOVA used to statistically test this trend are summarized in Table 2.

**Table 2:** Repeated measures ANOVA results for speech anxiety.

**Source**	***F*_(1, 37)_**	***p***	***ηp* ^2^**	**Interpretation**
Time	44.94	< 0.001	0.54	Significant main effect
Group	11.72	< 0.001	0.41	Significant main effect
Time × Group	26.33	< 0.001	0.42	Significant main effect

The repeated measures ANOVA results in Table 2 show that the main effect of time is significant [*F*_(1, 37)_ = 44.94, *p* < 0.001, η*p*^2^ =0.54]. This indicates a significant change in participants' speech anxiety levels over time, regardless of group, with a large effect size. The main effect of group is also significant [*F*_(1, 37)_ = 11.72, *p* < 0.001, η*p*^2^ =0.41], indicating that the overall speech anxiety levels of the experimental and control groups differ significantly, also with a large effect size.

The time × group interaction, which is central to the study's main hypothesis, is significant [*F*_(1, 37)_ = 26.33, *p* < 0.001, η*p*^2^ ≈ 0.42]. This interaction shows that the pattern of anxiety change over time in the experimental group participating in Instagram-based conversation activities differs significantly from that in the control group using traditional methods, with a large effect size. A marked decrease in speech anxiety was observed in the experimental group, while no significant reduction occurred in the control group. These results indicate that the Instagram-based learning environment is an effective tool for reducing Turkish speech anxiety among foreign students, and that the change over time was especially pronounced in the experimental group.

### 3.2 Findings related to the second research question

**Figure 6:**
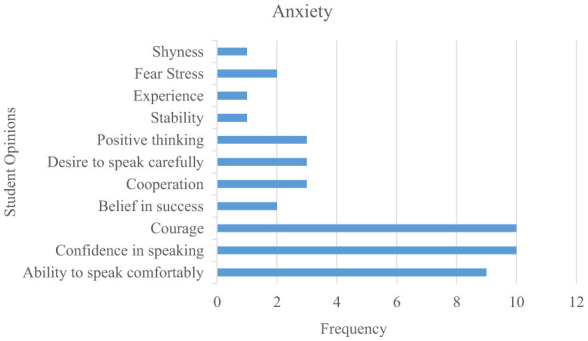
Student opinions on Instagram shared tasks related to speech anxiety.

The findings regarding the EG students' views on the contribution of Instagram shared conversation tasks to their speech anxiety and speech skills are shown in Figure 6.

When examining Figure 6, student responses to Instagram shared speaking tasks related to Turkish speech anxiety are largely concentrated in the categories of “Courage,” “Confidence in speaking,” and “Ability to speak comfortably.” It is evident that students generally approached these tasks positively and that collaboratively completed tasks provided affective benefits such as positive thinking, a desire to speak carefully, and belief in success. Overall, the findings indicate that students viewed Instagram shared tasks as an effective tool for reducing their Turkish speech anxiety.

Some of the EG students' views on their Turkish speaking anxieties regarding Instagram shared conversation activities are as follows:

*S11. Knowing that others were watching me speak Turkish made me a little nervous, but I was proud of my achievement. Overall, it was a rewarding experience that boosted my confidence*.

*S10. My pronunciation improved as I spoke with Turks during interviews. I now feel more confident when speaking Turkish*.

*S4. I can now speak comfortably with a Turk; I am no longer afraid*.

*S3. It was generally beneficial, especially the collaboration*.

*S9. I now feel better when talking to Turkish people*.

*S6. These interviews helped alleviate my anxieties. The more interviews I do, the better I feel*.

*S5. It helped us gain more self-confidence*.

**Figure 7:**
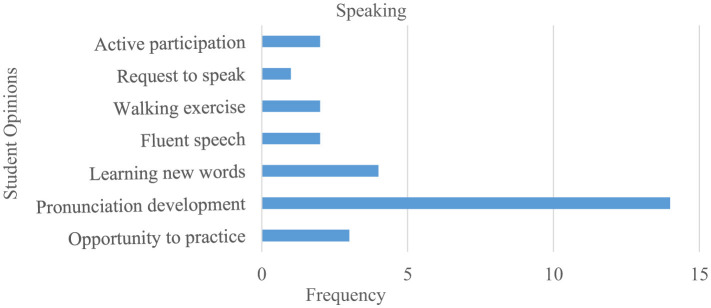
Student opinions on Instagram shared tasks for speaking skills.

Students' views on how Instagram-shared assignments improve their speaking skills are presented in Figure 7.

Figure 7 shows that students believe publishing speaking tasks on Instagram positively affects their Turkish speaking skills, particularly in pronunciation development, learning new words, and providing opportunities to practice with native speakers. Furthermore, students indicate that this application enables them to actively participate in speaking tasks and achieve gains such as fluent speaking.

Some participants' opinions regarding their speaking skills are as follows:

*S7. This application was somewhat beneficial for me. My pronunciation improved, and I think it was a good application*.

*S9. It helped me speak Turkish fluently*.

*S12. It helped me correct my pronunciation and enabled me to chat comfortably with a Turkish person*.

*S16. Interacting with native Turkish speakers allowed me to practice real-life conversations*.

*S2. In some videos, I wish I had spoken a little more because it greatly improved my Turkish speaking skills*.

**Figure 8:**
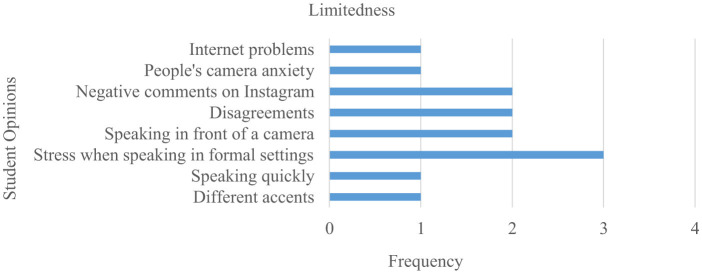
Limitations of Instagram shared tasks.

The limitations of the implementation process of Instagram shared tasks as reflected in student opinions are presented in Figure 8.

Figure 8 shows some limitations experienced within the application, based on students' opinions. Students indicate that Instagram shared conversation tasks cause at least some stress when speaking in formal settings. Reluctance to be interviewed and comments made on Instagram posts also contribute to a somewhat negative perception among students.

Participants also mentioned some limitations of the application in their feedback:

*S1. Some people felt uncomfortable speaking in front of the camera. We did not interview these individuals*.

*S6. It was difficult to convince people to participate in interviews*.

*S17. I was afraid that some people on Instagram would find my speech funny and might even write negative comments*.

*S10. At first, speaking Turkish in front of the camera was a bit difficult for me, but I got used to it*.

*S11. Being interviewed at university or in formal settings made me very nervous*.

### 3.3 Integration of quantitative and qualitative findings

**Figure 9:**
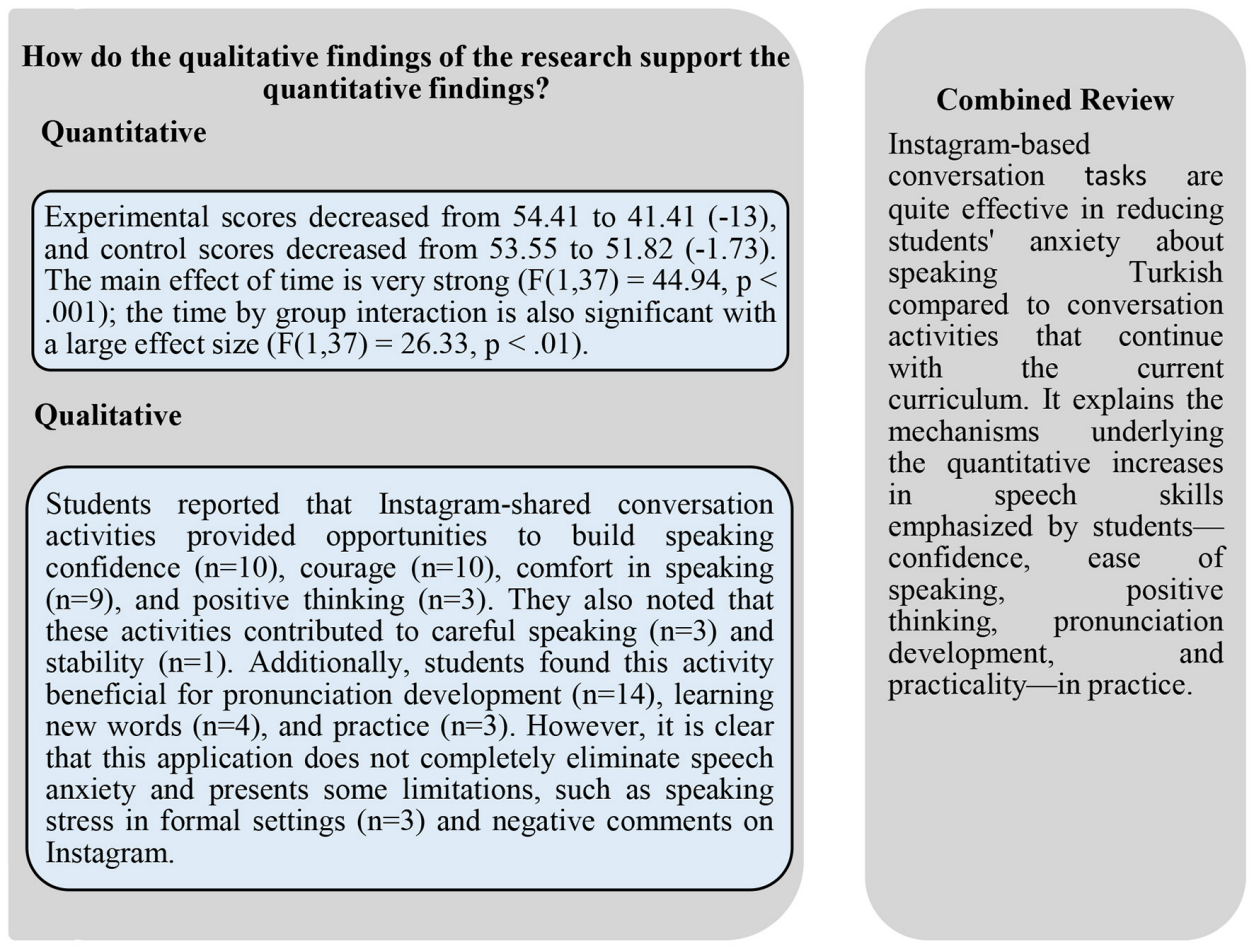
Integrated summary of the quantitative and qualitative findings of the research.

This section integrates and interprets the qualitative findings to support the quantitative results. The quantitative findings from the EG-CG group's comparison of speech anxiety related to Instagram shared conversation tasks and the qualitative findings from student experiences and opinions have been combined and summarized in a single presentation (Figure 9).

Figure 9 shows that quantitative results largely align with qualitative themes. Instagram shared task activities have strong positive effects on both reducing students' speech anxiety and supporting the development of speaking skills. Students describe these effects in terms of increased courage, speaking practice, pronunciation improvement, and greater self-confidence. However, students also clearly identified limitations of this application, such as stress in formal settings and negative comments. This indicates that Instagram shared speaking tasks are both a high-potential support tool and a risk area that must be carefully managed regarding speaking skills and anxiety in the TFL learning process.

## 4 Discussion

This study examined the effect of Instagram-shared speaking task activities on the speaking anxiety and speaking skill development of B2-level students within the TFL framework, using a mixed-methods (explanatory sequential) design. The quantitative findings show that the EG, who participated in Instagram-shared speaking tasks, had a statistically significant advantage over the CG, who performed speaking activities based on the current curriculum, in both speaking anxiety and speaking skill development levels. This advantage in speaking anxiety was evident in the EG, with a significant decrease from pre-test to post-test (x = 54.41 to x = 41.41), while the change in the CG remained limited (x = 53.55 to x = 51.82). Repeated measures ANOVA results indicated that the time × group interaction was significant [*F*_(1, 37)_ = 26.33, *p* < 0.001; η*p*^2^ ≈ 0.42], indicating a strong effect in favor of Instagram-shared speaking tasks. These quantitative findings are supported by qualitative data, specifically students' opinions, which reveal their experiences during the Instagram-shared conversation tasks. These results support the global literature on the effectiveness of Instagram-shared conversation tasks with strong empirical evidence in the TFL context; however, they are consistent with current discussions emphasizing that it is a tool requiring careful management during implementation (Aloraini, 2018; Parangu and Sulistyowati, 2020; Rizal and Farikhah, 2021).

The quantitative finding of the study is that EG students, who conducted their speaking classes using Instagram shared tasks, experienced a significant decrease in their speaking anxiety levels at the end of the study compared to CG students, who conducted their classes using speaking activities in the learning environment, as measured at the beginning of the study. This decrease in anxiety can be explained not only as a descriptive “adaptation” but also by a series of psychological mechanisms triggered by Instagram sharing tasks working together. The reasons underlying the decrease in speaking anxiety in EG can be explained by psychological mechanisms encountered by individuals in the process of acquiring foreign language speaking skills. In this study, the Instagram video-sharing tasks enabled students to engage in conversations with native Turkish speakers in real life, and the systematic repetition of these activities ensured that desensitization and exposure mechanisms were effective in reducing speaking anxiety. This result is consistent with the findings of similar studies in the literature (Duff et al., 2007; Finn et al., 2009). Qualitative findings directly reveal this process: The statement, “The more interviews I do, the better I feel” (S6), shows that repeated exposure reduces anxiety over time and makes speaking more manageable.

The use of Instagram, a social media application, as a tool to reduce speaking anxiety in TFL learners and its effectiveness aligns with recent studies on the impact of social media applications in supporting foreign language teaching and reducing students' foreign language anxiety (Chappell, 2014; Mondahl and Razmerita, 2014; Roohani and Heidari Vincheh, 2023). Similarly, (Zhao et al. 2022) found that using social media in the foreign language teaching process reduced participants' anxiety levels. In the study, EG students' views on Instagram shared task activities—which are effective in reducing speaking anxiety, particularly in pronunciation development, learning new words, comfort in speaking, gaining self-confidence, and courage—are also consistent with findings in the literature (Reinhardt, 2019; Aloraini and Cardoso, 2022; Yeh and Swinehart, 2022). However, these themes can also be interpreted at the mechanism level: pronunciation development and fluent speaking (S7, S9, S12) created an experience of “perceived competence” in students, strengthening their belief in self-efficacy. This strengthening accelerated the reduction of speaking anxiety. For example, the statement “My pronunciation improved as I spoke with Turks during interviews… I gained more courage” (S10) clearly shows the chain of mechanisms through which the acquisition of competence (pronunciation) is transformed into affective output (courage or decreased anxiety). The students' ability to freely choose their speaking environments with Turkish individuals has enabled them to feel more comfortable while speaking. This result aligns with the findings of similar studies (Zhang, 2010; Wu et al., 2025). EG students reported that repeating activities and engaging in real-life conversations with native Turkish speakers increased their self-confidence, which in turn positively affected their speaking anxiety. Studies demonstrating an inverse relationship between self-confidence and speaking anxiety (Toubot et al., 2018; Shamsuri et al., 2021; Quvanch et al., 2024) support this finding. DG students stated that they felt good about themselves due to the video-sharing activity tasks on Instagram. Based on these student perspectives, it can be inferred that their Turkish speaking self-efficacy has increased. This is because studies have shown that individuals with high foreign language speaking self-efficacy positively impact academic achievement and experience lower anxiety (Putwain et al., 2013; Zhen et al., 2017; Hayat et al., 2020). In this context, the quantitatively determined decrease in anxiety and qualitative themes (self-confidence, courage, comfortable speaking) can be explained together through the “self-efficacy development” mechanism.

The research is consistent with Aloraini and Cardoso's (2022) finding that social media facilitates language practice and the acquisition of new vocabulary in foreign language learning. Furthermore, the study's findings align with those of Ceh and Benedek (2025), who found that Instagram supports creative behavior and increases users' daily creative activities, providing an environment for users to express themselves and produce creative content. In this respect, Instagram offers not only a practical space but also a learning ecosystem that nurtures students' self-efficacy through the experience of expressing themselves and producing visible products.

The study found that TFL students improved their speaking skills during the foreign language learning process by completing assigned tasks, as shown by student feedback. This result aligns with the findings of (Göçer and Karadag 2020), who concluded that task-based teaching methods help individuals become proficient in the language used in daily life. Consistent with the principle of task-based foreign language learning, which is the theoretical basis of the study, students made Instagram posts as part of the assigned tasks, and this had a positive effect on their speaking anxiety. Student statements (e.g., S16: “it allowed me to practice real-life conversations”; S2: “it greatly improved my Turkish speaking”) indicate that the task-based application strengthened the “real-life practice” component and accelerated the exposure and practice process that reduces anxiety.

Research indicates that Instagram, as a social media tool, contributes positively to TFL students' speaking skills and learning environments. Their reflections on gains such as collaboration, self-confidence, and active participation also support current research on social media in foreign language teaching (Lampe et al., 2015; Eid and Al-Jabri, 2016; Güleç and Güleç, 2017; Kukulska-Hulme and Viberg, 2018; Raza et al., 2020; Ngoc Hoi, 2023; Reinhardt, 2019). Additionally, the fact that Instagram-shared conversation tasks allow EG students to share experiences such as motivation and extracurricular activities indicates that this application can help prepare suitable environments and activities for foreign language learners and teachers. In this regard, the findings of this study align with Yilmaz's (2021) research, which concluded that students experience positive outcomes from using social media in Turkish language teaching processes. Additionally, these positive outcomes are similar to the results of studies on social media use in EFL contexts (Rosell-Aguilar, 2018; Ismail et al., 2019; Listiani et al., 2021; Barrot, 2020, 2022). Another important finding is that DG learners acquired new words and expanded their vocabulary by communicating with speakers of the target language through the tasks. Similarly, studies have found that Telegram, another social media tool, supports EFL learners' vocabulary development and word learning (Zarei et al., 2017; Abu-Ayfah, 2020). Aşar and Özdemir's (2024) study, which found that Instagram's interactive and visual structure enhances learning motivation and vocabulary development in foreign language teaching, is consistent with the findings of this research. The results also align with (Gücüyeter et al. 2019), who concluded that TFL students' social media interactions positively affect performance by creating a rich learning environment through social activities. In the qualitative themes, “learning new words,” “pronunciation development,” and “fluent speaking” (Figure 7; S7, S9, S12) not only support skill acquisition but also reinforce students' “I can do it” perception, thereby reducing anxiety and supporting the self-efficacy mechanism.

Although Instagram-based shared tasks positively affect TFL students' speaking anxiety levels and influence their speaking skills, research findings also reveal that students experience speaking anxiety in formal settings, encounter internet problems, and receive negative comments. The fear of being ridiculed or criticized by peers causes students to become shy and anxious (Kankam and Boateng, 2017). Although the fear of negative evaluation by peers initially had a negative impact on the speaking anxiety of EG students, student feedback indicates that they overcame this negativity in subsequent activity repetitions. This situation can be explained by the mechanism of “decreased assessment threat”: students initially perceived the presence of cameras or being watched as a threat (S11; S17), but as tasks were repeated, they became accustomed to this and their anxiety decreased (S10: “then I got used to it”). The support students received from peers when working together, participating in activities, and sharing activity videos on Instagram may have contributed to these perceptions. The statement “it was nice to collaborate” (S3) suggests that peer-supported interaction may have reduced the threat of evaluation by increasing feelings of social security. This result aligns with similar studies, which show that exposure over time leads to habituation to anxiety-inducing social situations, resulting in a gradual decrease in anxiety levels (Özgür, 2017; Yadav et al., 2019; King, 2016; Li and Wang, 2026). Therefore, the quantitatively observed strong decrease in anxiety can be interpreted as an integrated outcome of the reported qualitative findings: (i) repetition/exposure-habituation, (ii) increased self-efficacy and perceived competence, (iii) reduced evaluation threat, and (iv) peer-supported interaction processes working together.

This integrated explanation strengthens the theoretical contribution of the study by clarifying the effect of Instagram shared conversation tasks not only at the outcome level but also at the level of psychological processes that lead to anxiety reduction and conversation development (exposure and desensitization, self-efficacy development, reduction of evaluation threat, and peer-supported interaction).

## 5 Conclusion

The effective use of social media in foreign language teaching and its integration into education align with contemporary learning approaches and technological developments. In this context, the research has revealed important findings about activities conducted on Instagram within the broader scope of social media and their impact on students' speech anxiety and the development of their speaking skills. The quantitative results show that speech anxiety decreased significantly in the experimental group that participated in Instagram-based speaking tasks, while the decrease was limited in the control group. This study offers implications for teaching and provides insights into rapidly growing social media trends, technological innovations, and their creative uses for language learning.

This mixed-methods study examined the integration of Instagram as a complementary tool in TFL students' speaking activities and statistically identified changes in students' speaking anxiety levels. These quantitative results were supported by student feedback describing their experiences with the application, and the research findings were interpreted through integration of both data sources. This integrated assessment explains the effect of Instagram-shared speaking tasks not only at the outcome level but also at the level of psychological processes that contribute to anxiety reduction and speaking development. The study revealed that certain psychological mechanisms are effective factors in reducing speaking anxiety among EG students in this context. These mechanisms can be organized as follows: (i) exposure, practice, and desensitization through repetitive tasks; (ii) perceived competence and self-efficacy in Turkish speaking, strengthened by progress in pronunciation and fluency; (iii) gradual reduction of fear of negative evaluation over time; and (iv) collaborative or peer-supported interaction. Understanding these factors is important for educators and researchers in developing effective strategies to alleviate speech anxiety among TFL learners. In this context, designing tasks with a repetitive structure, providing feedback in a safe environment, and planning collaborative activities that increase peer support can be considered pedagogical implications that may enhance the anxiety-reducing effect.

Although empirical studies integrating social media tools into foreign language teaching exist in the literature, the lack of comprehensive research on Instagram specifically addressing speaking anxiety in the TFL context makes this study a valuable resource for interested readers. Furthermore, the study highlights both the possibilities and limitations of this Instagram-based application, which has attracted considerable interest from foreign students learning Turkish in recent years. Student feedback indicates limitations such as the stress caused by speaking in formal settings and the potential for negative comments to increase evaluation anxiety. Therefore, while Instagram-shared speaking tasks offer significant support potential, they must be carefully managed with attention to these risk areas.

## 6 Future research and limitations

This study presents promising findings on integrating social media into foreign language teaching environments and its potential to reduce students' foreign language anxiety. It specifically highlights Instagram as a digital platform for sharing speaking activities within the TFL context, particularly for speaking skills. However, certain limitations of the study restrict its generalizability.

The study sample consists of B2-level TFL students at a single state university and is limited to a 6-week B2 course period in Turkey. Therefore, future studies could be conducted over a longer period, covering more generalizable language class levels, different languages, and other countries. The study is also limited to Instagram among social media tools and to speaking among language skills. The positive effects of students' Instagram-shared speaking activities on their speech anxiety do not indicate that this tool directly serves this purpose; rather, positive results may arise from students experiencing a new and different learning process. More positive and generalizable results may emerge by testing different social media tools and language skills. The findings reveal that Instagram-shared speaking activities offer cognitive and affective opportunities for TFL students when combined with appropriate pedagogical design, instructor guidance, and critical social media use.

## Data Availability

The original contributions presented in the study are included in the article/Supplementary material, further inquiries can be directed to the corresponding author.
