# Supplementary material for: Instagram shared tasks in reducing speech anxiety among international students learning Turkish
Source: Front Psychol. 2026 Mar 17;17:1775400. doi: 10.3389/fpsyg.2026.1775400 (PMC13037416; doi:10.3389/fpsyg.2026.1775400)
Supplement: Supplementary file 1 [file Data_Sheet_1.docx]

**Appendix 1**

**Event Implementation Guidelines** (English version)

**Publishing Speech Events on Social Media: The Instagram Example**

**5. Week -** Subject: Discussion about Technology

• First, each group will conduct an interview with a Turkish person about “Technology.”

• In this interview, each member of the group will conduct an interview with another member, and this interview will be recorded on camera by the other member. (The recording will be at least 2 minutes long.)

• Information about the interview will be provided before it begins. (You can start by introducing yourself and explaining why you are conducting this interview.)

(Example: Hello, we are students here at the university. I am from Guinea and my friend is from Congo. We would like to conduct a short interview with you about “Social Media.” Do you have permission? Thank you.)

**Interview Questions**

- Do you think the rapid advancement of technology is beneficial or harmful to humanity? Why?
- So, how does the advancement of technology affect you? (in your work or education)
- What technological products do you use?
- What do you look for when buying a technology product? (quality, price, usability, etc.)
- Would you like to live in a time without technology? Why?
- Do you agree with the idea that “Technology makes people lazy”? Why?
- Do you think technological development in Turkey is sufficient? In which areas would you like to see further development?
- What was the last technological product you purchased? Where did you make this purchase?
- I also want to buy a technological product. Which product would you recommend to me?
- Thank you for your participation and feedback. Have a nice day…

• You can do this activity outside of class, on campus and in the city center.

• Recorded videos will be posted on the TÖMER Instagram account by Monday at the latest.
