# Supplementary material for: Instagram shared tasks in reducing speech anxiety among international students learning Turkish
Source: Front Psychol. 2026 Mar 17;17:1775400. doi: 10.3389/fpsyg.2026.1775400 (PMC13037416; doi:10.3389/fpsyg.2026.1775400)
Supplement: Supplementary file 2 [file Data_Sheet_2.docx]

**Appendix 2.**

**Semi-Structured Interview Guide and Application Protocol** (English version)

Research: Instagram Shared Tasks in Reducing Speech Anxiety Among International Students Learning Turkish

Participant Code: ……… Date: ………… Duration (min): ……… Interviewer: …………

**1) Standard pre-interview briefing (2–3 min)**

Standard text to be read:

“We would like to understand your experiences with the after-class interview activity and Instagram post. Your answers will only be used for academic purposes; no name/identity information will be shared. You may choose not to answer any question or end the interview at any time. There are no right or wrong answers; only your experience matters.”

Record/Note:

☐ Audio recording taken ☐ Audio recording not taken (note only) ☐ Consent obtained

**2) Warm-up questions (1 min)**

• “How were your Turkish speaking exercises in general this term?”

• “How often do you use Instagram in your daily life?” (short)

**3) Main questions (asked in the same order)**

Application rule: Each participant is asked the questions in the same order. If the response is short/closed, only neutral probing questions are used; leading statements are avoided.

Q1. How did the interview activities affect your anxiety about speaking Turkish? (decreased/increased/remained the same) Please explain.

Neutral probing questions:

• “Can you give me an example?”

• “When did you feel it most?”

• “What changed before and after the event?”

Q2. What were the limitations/challenging aspects of the activities?

a) “What was the most challenging situation/event you encountered during the activities?”

b) “How did you solve the problems you encountered during the activities?”

c) “In which situations did you experience anxiety about speaking Turkish during the activities? Please explain.”

Neutral probing questions:

• “How often did these difficulties occur?”

• “Where/with whom did they occur most often?”

Q3. Did you encounter any problems when posting/sharing event videos on Instagram? Please explain.

Neutral probing questions:

• “Was it a technical problem or content-related?”

• “Did this situation affect your speaking anxiety? How?”

Q4. What feelings and thoughts did posting your event videos on Instagram evoke in you? (e.g., excitement, anxiety, the thought that everyone would see it, the desire to speak more carefully, etc.)

Neutral probing questions:

• “Which emotion was more dominant? Why?”

• “How did the thought that everyone would see you affect your speech?”

• “Did you feel the need to speak more carefully? How?”

Q5. How did this activity contribute to your Turkish speaking skills? Explain.

Neutral probing questions:

• “Has there been a change in terms of fluency/pronunciation/word choice/confidence?”

• “Has it affected your desire to use Turkish outside of class?”

**4) Closing the meeting (1–2 min)**

• “Is there anything else you would like to add?”

• “Do you have any suggestions for improving this event?”

Thanks: Thanks for participating; if there is a recording, it is turned off.

**5) Interviewer notes (after the interview, 1 min)**

• Points of interest (brief): …………………………………………………………………

• Notes on the flow of the interview (brief): ……………………………………
